# The Complementary Effects of Atorvastatin and Exercise Treatment on the Composition and Stability of the Atherosclerotic Plaques in ApoE Knockout Mice

**DOI:** 10.1371/journal.pone.0108240

**Published:** 2014-09-29

**Authors:** Petros Moustardas, Nikolaos P. E. Kadoglou, Michalis Katsimpoulas, Alkistis Kapelouzou, Nikolaos Kostomitsopoulos, Panayotis E. Karayannacos, Alkiviadis Kostakis, Christos D. Liapis

**Affiliations:** 1 Center for Experimental Surgery, Biomedical Research Foundation, Academy of Athens, Athens, Greece; 2 Department of Vascular Surgery, «Attikon» Hospital, Medical School, University of Athens, Athens, Greece; Brigham and Women's Hospital, Harvard Medical School, United States of America

## Abstract

**Aim:**

This study aimed to investigate the effects of combined atorvastatin and exercise treatment on the composition and stability of the atherosclerotic plaques in apolipoproteinE (apoE) knockout mice.

**Methods:**

Forty male, apoE^−/−^ mice were fed a high-fat diet for 16 weeks. Thereafter, while maintained on high-fat diet, they were randomized into four (n = 10) groups for 8 additional weeks: Group CO: Control. Group AT: Atorvastatin treatment (10 mg/Kg/day). Group EX: Exercise-training on treadmill. Group AT+EX: Atorvastatin and simultaneous exercise training. At the study’s end, plasma cholesterol levels, lipids and triglycerides were measured, along with the circulating concentrations of matrix-metalloproteinases (MMP-2,3,8,9) and their inhibitors (TIMP-1,2,3). Plaque area and the relative concentrations of collagen, elastin, macrophages, smooth muscle cells, MMP-2,3,8,9 and TIMP-1,2,3 within plaques were determined. Lastly, MMP activity was assessed in the aortic arch.

**Results:**

All intervention groups showed a lower degree of lumen stenosis, with atheromatous plaques containing more collagen and elastin. AT+EX group had less stenosis and more elastin compared to single intervention groups. MMP-3,-8 -9 and macrophage intra-plaque levels were reduced in all intervention groups. EX group had increased TIMP-1 levels within the lesions, while TIMP-2 was decreased in all intervention groups. The blood levels of the above molecules increased during atherosclerosis development, but they did not change after the therapeutic interventions in accordance to their intra-plaque levels.

**Conclusion:**

The two therapeutic strategies act with synergy regarding the extent of the lesions and lumen stenosis. They stabilize the plaque, increasing its content in elastin and collagen, by influencing the MMP/TIMP equilibrium, which is mainly associated with the macrophage amount. While the increased MMP-2,-3,-8 -9, as well as TIMP-1 and TIMP-2 circulating levels are markers of atherosclerosis, they are not correlated with their corresponding concentrations within the lesions after the therapeutic interventions, and cannot serve as markers for the disease development/amelioration.

## Introduction

Atherosclerosis and its complications constitute the predominant cause of death worldwide [1]. Up today, regression of the atherosclerotic lesions remains the gold standard of most pharmaceutical or interventional therapeutic strategies. Alternatively, a growing body of evidence outlines the clinical importance of atherosclerotic lesions’ composition [2]. In particular, changes in the atherosclerotic plaque composition rather than the percentage of lumen narrowing seem to predominantly influence the clinical outcomes and prognosis of atherosclerosis. Plaque composition is closely associated with traditional (e.g. dyslipidemia, hypertension) and non-traditional (e.g. inflammatory markers, matrix-metalloproteinases-MMPs) cardiovascular risk factors [3], [4]. The modification of the latter risk factors has been increasingly suggested as the target of all therapeutic interventions.

HMG-CoA reductase inhibitors (statins) intervene early in the cholesterol synthesis pathway, by decreasing its plasma concentration [5]. Statin treatment has been well-documented to considerably reduce cardiovascular morbidity and mortality [6]. Notably, those overall benefits were disproportionally greater than those expected from the achieved improvement in lipid profile. The latter fact supports the notion of multiple, “pleiotropic” properties of statins, extending their efficacy beyond lipid-lowering [7]. Exercise, on the other hand, comprises another important therapeutic mainstay of cardiovascular diseases. It’s widely accepted that increased physical activity suppresses atherosclerotic-related morbidity and mortality rate in the general population [8]. Regarding the underlying mechanisms, the cardiovascular benefits derived from systemic exercise can be partly explained by the modification of traditional cardiovascular risk factors [9]. Similar to statins, physical activity may exhibit additional “pleiotropic” actions, which mostly remain elusive [9]. Taken together, the “pleiotropic” properties of the aforementioned therapeutic modalities, statins and exercise training, seem quite promising in cardiovascular disease prevention.

MMPs, an expanding family of zinc-dependent endopeptidases, exert proteolytic activity towards all components of the extracellular matrix [10]. Numerous experimental and clinical studies underline the key-role of MMPs in the atherosclerotic plaque development and rupture [11]–[13]. On the other hand, their endogenous inhibitors (Tissue Inhibitors of MMPs - TIMPs) actively participate in MMPs activity regulation [14]. A shift in MMPs/TIMPs equilibrium to MMPs prevalence will cause an increase in net proteolytic activity, contributing to plaque destabilization and a higher incidence of cardiovascular events and vice versa [15].

In the present study, we investigated the effects of combined treatment of exercise training and atorvastatin on plaque stability and the MMP/TIMP activity, using a valid animal model of atherosclerosis (apoE knockout mice). We hypothesized that the combined treatment would confer better results than each intervention alone, advocating for their “pleiotropic” effects against atherosclerosis.

## Materials and Methods

### Study design

Forty male mice with homozygous deficiency in apolipoprotein-E (apoE^−/−^, C57BL/6J background), aging 8 weeks, and weighting 20–25 g were fed a western-type high-fat diet (Harlan, Teklad; 88137) for 16 weeks, in order to develop atherosclerotic lesions. Thereafter, high-fat diet was maintained and all mice were randomized into four equivalent (n = 10) groups. Group CO: No therapeutic intervention was performed and served as control group. Group AT: Mice received Atorvastatin administration. Group EX: Mice underwent an exercise program. Group AT+EX (atorvastatin + exercise): Atorvastatin was administered as in group AT and concomitantly mice underwent an exercise program as group EX.

The study protocol was approved by the local ethics committee (Athens Prefecture Veterinarian Service; Κ/2953/23-4-2007) and took place in the animal facilities of the Center for Experimental Surgery of the Biomedical Research Foundation of the Academy of Athens. The animals were kept in a 12 hr light/12 hr dark environment under constant temperature of 21°C, grouped in cages of 5 individuals each and were handled in accordance to the relevant international guidelines for the proper care and use of laboratory animals.

### Therapeutic Interventions

Atorvastatin treated mice received 10 mg/Kg/day via esophageal gavage [16]. The administered aqueous suspension was freshly prepared, in the concentration of 1 mg/ml, so that each mouse received 10 µl of suspension per gram of its weight. Exercised mice were gradually accustomed to the training program’s daily duration and speed over the first training week (full program: 5 times/week, 40 min/session, velocity = 12 m/min, slope = 5°), which was accomplished in a motorized treadmill (Exer-6 M Open Treadmill, Columbus Instruments, USA) with 6 separate compartments, so that an equal amount of animals could train simultaneously in an individual manner. Furthermore, stimuli on the treadmill were mostly mechanical, and electrical shock-plate was only used during the first session, and only in some individual mice, for them to get accustomed to the training procedure. During the exercise sessions, the mice were closely monitored for the verification of successful session completion. The animals that did not receive any of the two treatment modalities for the duration of the interventions, received the corresponding placebo treatment which included esophageal injection of 100 µl PBS, for non-atorvastatin receiving animals, and daily placement on the stable treadmill for 40 min for non-exercising animals.

### Tissue collection and processing

After overnight fasting, blood samples were obtained, under anesthesia (inhaled isoflurane), at the study entry via gastrocnemial muscle puncture and the end, via cardiac puncture. Fasting glucose, triglyceride and cholesterol levels were assessed. At the end of the study (24^th^ week), all mice were sacrificed. Before euthanasia, body weight was measured and blood samples were taken. During euthanasia, the heart along with the aorta was perfused with a total volume of 1 ml 10% neutral buffered formalin solution via heart puncture. Afterwards, they were removed and maintained in a 10% neutral buffered formalin solution for 24 hours. Subsequently, they were embedded in paraffin blocks with exact vertical orientation of the aorta. Parts of the aortic arch were dissected and snap-frozen for the subsequent zymographic analysis.

### Histology

**Figure 1:**
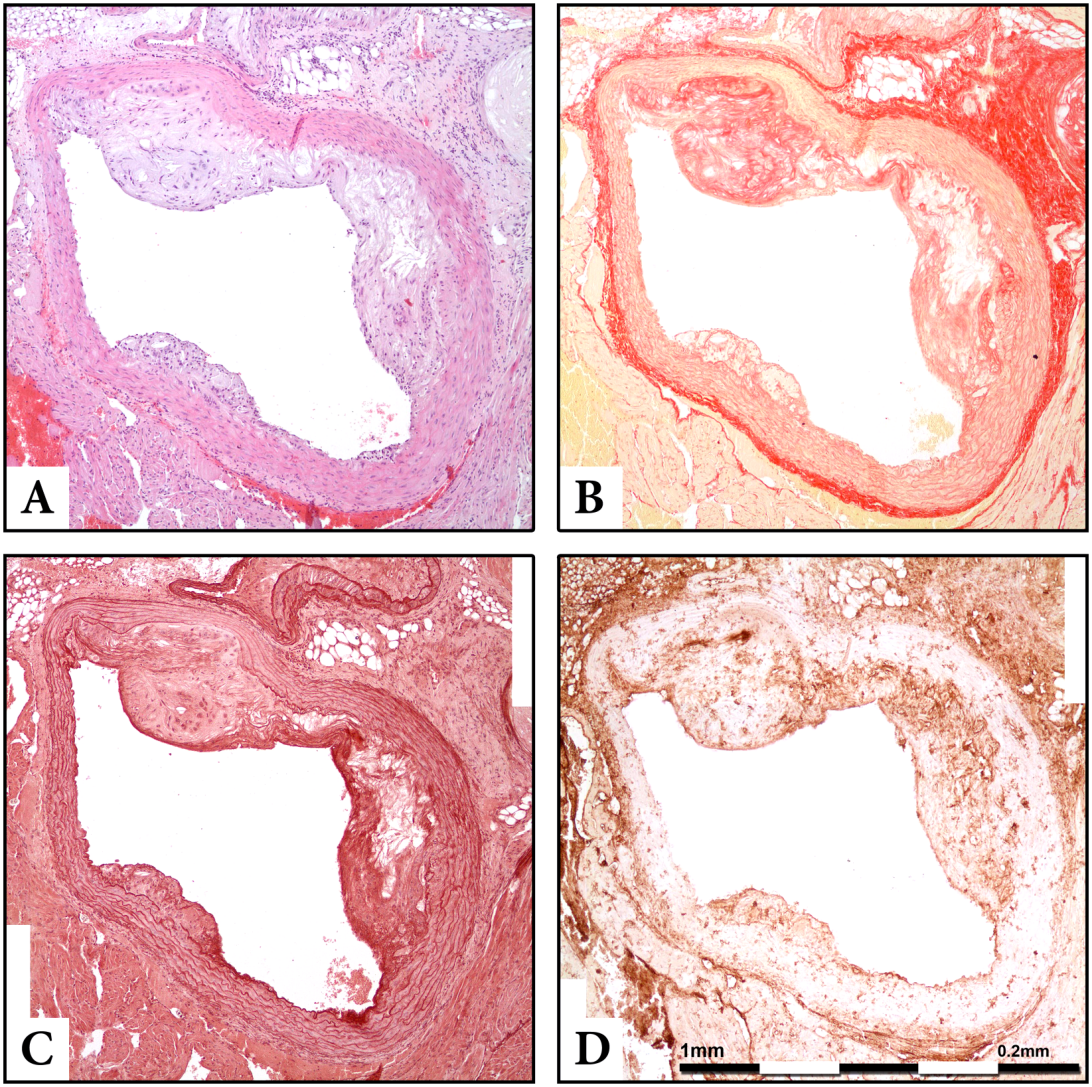
Co-localized stains of serial sections of the aortic arch in Apo-E−/− mouse. Section thickness was set at 5 µm and original magnification at 100x. A: Haematoxylin-Eosin stain for morphometry. B: Sirius red for collagen stain. C: Orcein for elastin stain. D: Immunohistochemistry, MMP-2 stain.

**Figure 2:**
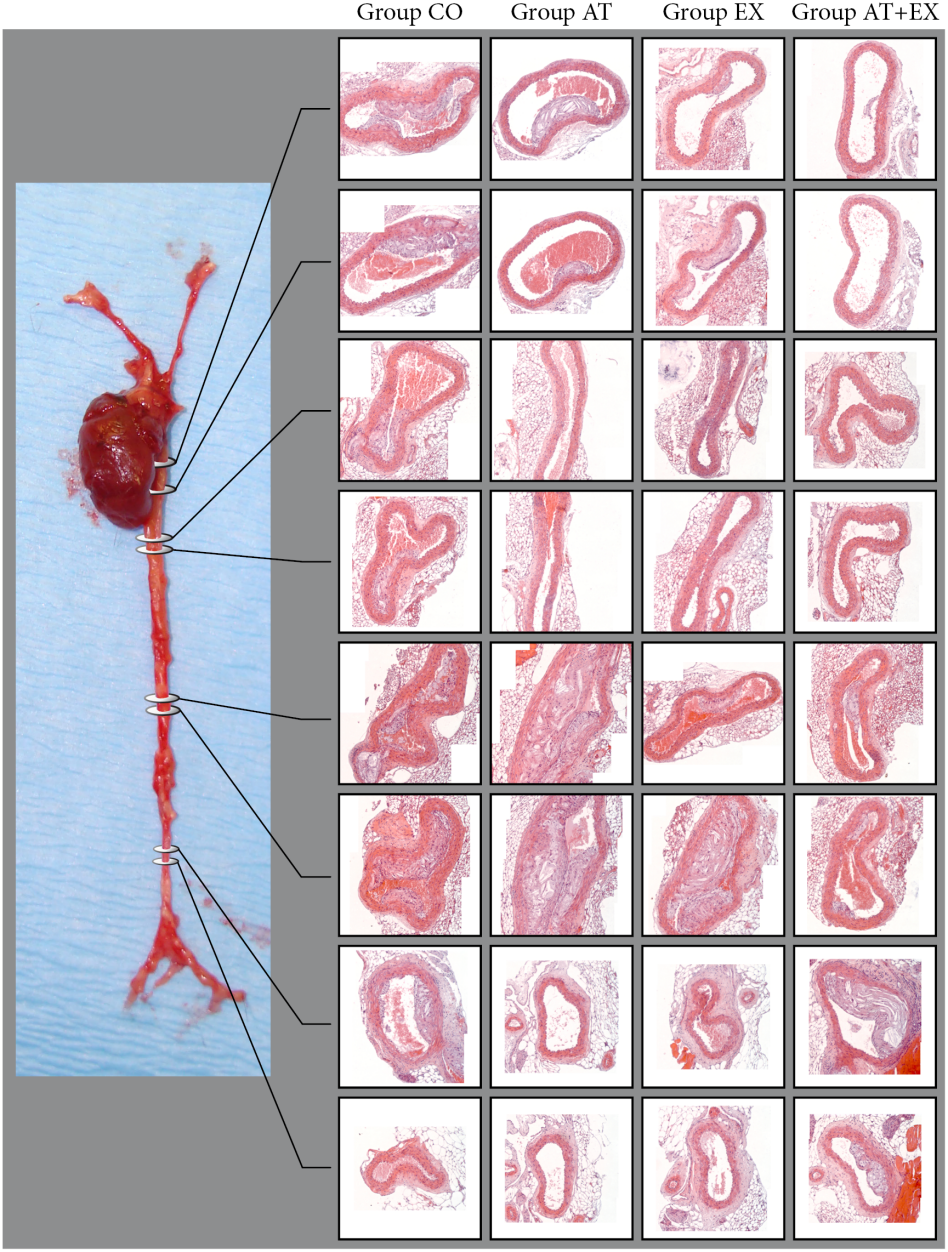
Representative images from whole aorta sectioning and H+E staining in predefined levels (Descending Thoracic Aorta in four levels, Abdoninal Aorta above renal arteries in two levels and Abdominal Aorta below Renal Arteries in two levels. Original magnification: 200x.

In a microtome, serial sectioning started from the aortic valve leaflets and proceeded across the aortic arch, with section thickness set at 5 µm and following a standardized protocol which allowed the co-localization for the various measured variables (Figure 1). Slices were collected on poly-D-lysine-coated slides. Progressive hematoxylin/eosin (H&E) stain was used for morphometrical purposes (Figures 1, 2). In particular, 10 sections per mouse, at equal (50 µm) intervals over a distance of 500 µm of the aortic arch, were taken and stained with H&E for quantitative morphometric analysis. The first section of each sequence was at a distance of 50 µm after the aortic valve. Additionally, the thoracic aorta, as well as the abdominal aorta was sectioned in predefined positions, followed by the H&E stain and morphometric analysis (Figure 2).

**Figure 3:**
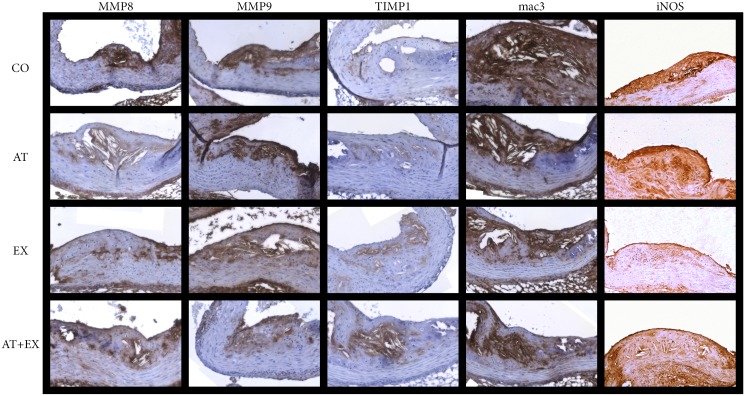
Immunohistochemical staining with antibodies against MMP-8, MMP-9, TIMP-1, mac-3 and iNOS in optical microscopy sections of experimental groups CO, AT, EX and AT+EX. Section thickness was set at 5 µm and original magnification at 100x.

Sirius Red staining allows to identify collagen distribution within the aortic sections, while Orcein and Verhoeff’s Van Gieson (EVG) stain visualize elastin content (Figures 1, S1, S2). By means of immunohistochemistry we used antibodies corresponding to MMP-2, MMP-3 MMP-8, (MBL, International Corporation, Woburn, MA, USA), MMP-9 (AbD Serotec, Oxford, UK), mac-3 antigen of murine macrophages (BD Pharmigen, Franklin Lakes, NJ, USA), alpha-smooth muscle isoform of actin (Biocare Medical, LLC, Concord, CA, USA), TIMP-1 (Triple Point Biologics Inc., Forest Grove, OR, USA), TIMP-2 (Acris Antibodies GmbH, Hiddnhausen, Germany), TIMP-3 (Abcam, Cambridge, UK), eNOS and iNOS (Assay Designs, Ann Arbor, MI, USA) for the staining of the corresponding antigens. Similarly to H&E procedure, 6 sections per mouse at equal intervals were stained with the aforementioned substances, respectively (Figures 3, S3, S4). The whole procedure of specimen and data collection was blinded to the researchers.

### Digital processing - Histomorphometric analysis

All sections were observed under 100x magnification in an Olympus microscope, (CX31, Hertfordshire, Olympus UK Ltd) and digital pictures were acquired and stored in a lossless format using a “U-TVO, 5xC-3, N1512100” microscope camera and “Altras Soft Imaging System” computer software.

Image analysis software (Image Pro Plus Version 4.1; Media Cybernetics) was used for morphometric image analysis. The extent of the atherosclerotic plaques (in µm^2^) was measured and the total lumen area (in µm^2^) was calculated by measuring the internal elastic lamina (IEL) perimeter and extrapolating the circumscribed area for each section. More specifically, while we could not measure the lumen area directly using images of non-perfectly circular aortic cross-sections, we assessed it indirectly, by measuring the internal perimeter of the lumen which remains unchanged regardless of the tissue folding. We then derived the circular lumen area using the following perimeter to area formula: A = P^2^/4π, where A = the lumen area and P = the measured perimeter, π = the mathematical constant of the ratio of a circle’s circumference to its diameter. Again, the IEL distinguished plaque from arterial wall. The proportion of calculated total lumen area occupied by the atherosclerotic plaques expressed the percentage of luminal stenosis, in each section. For atherosclerotic lesion quantification, we averaged plaque and lumen area and the luminal stenosis from all hematoxylin/eosin-stained sections per animal. For the measurement of the relative concentrations of the stained molecules, the segmental stained plaque area was expressed as percentage of the whole atherosclerotic plaque area [17].

### Immunosorbent Assay

For the assessment of the circulating levels of the examined MMPs and TIMPs, blood serum samples acquired both at the study’s entry and the study’s end were analyzed by means of sandwich ELISA. Specifically, we measured the concentrations of MMP-2, MMP-3, MMP-8, MMP-9, TIMP-1 (Corresponding ELISA kits, R&D Systems, Minneapolis, MN, USA) as wells as those of TIMP-2 and TIMP-3 (USCN Life Science Inc., Houston, TX, USA). Serum samples for the assessment of MMPs were used at a dilution of 1/20, while those for the assessment of TIMPs were diluted at 1/4. All samples and standard curve dilutions were measured in doubles, and a mean value was calculated.

### Zymography

The semi-quantitative assessment of the MMP and TIMP activity was performed by means of SDS-PAGE zymography, in 10% gelatin zymogram gels (Invitrogen – Life Technologies, Carlsbad, CA, U.S). MMP activity was measured directly via densitometry (ImageJ software v.1.46, National Institutes of Health, USA) whereas for TIMP activity, reverse zymography was carried out, using MMP-2 as enzyme substrates and gelatin as the MMP-2 substrate. The digital negatives of the final images from reverse zymography were produced in order to be measured via densitometry. Individual MMPs and TIMPs were identified by their molecular weight, using a protein molecular weight standard (Invitrogen – Life Technologies, Carlsbad, CA, USA).

### Statistical analysis

The results of the study are presented as mean values ± Standard deviation (S.D.). Normality of distribution was assessed using the Shapiro-Wilk test. The comparisons between and within groups were performed using Student’s t-test and paired samples t-test respectively, while a comparison of all four groups was made using one way ANOVA and post-hoc Tuckey HSD test. A two-tailed p value<.05 was considered statistically significant. All statistical analyses were based on SPSS v20.0 (IBM, Armonk, NY, USA).

Detailed methods and protocols of the various described experiments are presented in detail in the supplementary protocols file (Protocols S1).

## Results

Baseline and final values of variables are listed in Tables 1–4. There were no significant differences between groups at baseline. Importantly, all mice completed experimental procedures and therefore their data were included in statistical analysis. Body-weight showed a gradual increment during the 16-week high-fat feeding. Afterwards, we observed a significant weight-loss in all the study’s groups, as indicated by the paired-samples test (p values; CO: p = .043, AT: p = .003, EX: p<.001, AT+EX: p = .005). Thus differences in body-weight loss between groups did not reach significant level (p>.05). Glucose levels displayed a significant reduction only within group AT+EX. In parallel, atorvastatin-treated groups (AT and AT+EX) showed considerably lower total cholesterol values when compared to either control or exercise-treated groups (p<.05). Triglycerides plasma concentrations were also considerably lower in all atorvastatin treated groups compared to the control group (p<.05).

**Table 1:** Body-weight and plasma levels of glucose, total cholesterol, and triglycerides at the intervention start and the end of the study.

		Body weight (g)	Glucose (mg/dl)	Triglycerides (mg/dl)	Cholesterol (mg/dl)
**CO (n = 10)**	week 16	38.6±6.1	260±24.9		
	week 24	37.7±5.4	250.30±30.9	112±22	685.7±33.1
	P1	.043	.13		
**AT (n = 10)**	week 16	37±5.0	254.4±40.0		
	week 24	36.6±4.9	235.2±33.9	93.5±15.7	586.6±34.7
	P1	.003	.051		
	P2	.182	.368	.045	<.001
**EX (n = 10)**	week 16	39.4±4.1	264.5±23.7		
	week 24	37.9±3.4	253.2±32.5	99.7±7.4	650.4±44.5
	P1	<.001	.060		
	P2	.290	.868	.123	.060
**AT+EX (n = 10)**	week 16	39.1±3.9	258.5±25.3		
	week 24	38.0±3.5	237.3±22.8	89.7±9.6	537.1±47.9
	P1	.005	.011		
	P2	.729	.207	.012	<.001
**One Way Annova p values**	P3	.064	.526	.011	<.001
**Tuckey HSD post Hoc p values**	AT vs EX	.044	.818	.790	.006
	AT vs AT+EX	.247	.997	.940	.046
	EX vs AT+EX	.829	.700	.449	<.001

P1: p value of changes of variables within groups (paired samples t test); P2, p value of variable difference between week 16 and week 24 compared to variable difference of the control group (independent values t test); P3: significance of variable differences between all groups (one-way ANOVA).

Experimental week count started when mice were 8 weeks old, and entered high fat diet.

**Table 2:** Atherosclerotic plaque area, lumen area, lumen stenosis (H&E staining), intra-plaque elastin and tunica media elastin content (orcein staining), intra-plaque collagen (sirius red staining) and total average intra-plaque MMP/TIMP positive stain (IHC).

	CΟ (n = 10)	ΑΤ (n = 10)	EX (n = 10)	ΑΤ+EX (n = 10)	ANOVA, post-hoc p values
Aortic Arch PlaqueArea (x10^3^ µm^2^)	569.2±156.9	414.4±99.2	463.2±102.9	392.6±84.55	P_2_ = .776 P_3_ = .973 P_4_ = .519
		p = .017	p = .091	p = .007	P_A_ = .007
Aortic Arch LumenStenosis (%)	47.4±13.2	34.8±7.8	35.1±8.4	30.8±4.5	P_2_>.999 P_3_ = .760 P_4_ = .706
		p = .018	p = .023	p = .003	P_A_ = .001
Aortic Arch LumenArea (x10^3^ µm^2^)	1209.5±165.6	1198.8±142.7	1326.5±116.1	1275.7±177.2	P_2_ = .256 P_3_ = .674 P_4_ = .878
		p = .155	p = .084	p = .40	P_A_ = .220
Thoracic Aorta PlaqueArea (x10^3^ µm^2^)	119.0±93.3	102.4±84.6	94.8±90.3	93.5±85.6	P_2_ = .997 P_3_ = .996 P_4_>.999
		p = .683	p = .563	p = .532	P_A_ = .912
Thoracic Aorta LumenArea (x10^3^ µm^2^)	868.4±105.4	844.5±120.7.0	925.9±123.2	872.5±132.0	P_2_ = .444 P_3_ = .954 P_4_ = .757
		p = .644	p = .277	p = .939	P_A_ = .497
Abdominal Aorta level1 Plaque Area (x10^3^ µm^2^)	758.6±192.6	530.8±123.7	587.7±125.5	473.4±126.8	P_2_ = .817 P_3_ = .812 P_4_ = .308
		p = .006	p = .030	p = .001	P_A_ = .001
Abdominal Aorta level1 Lumen Area (x10^3^ µm^2^)	1563.8±238.6	1521.9±214.6	1722.8±211.0	1616.5±265.3	P_2_ = .236 P_3_ = .802 P_4_ = .740
		p = .685	p = .132	p = .646	P_A_ = .264
Abdominal Aorta level2 Plaque Area (x10^3^ µm^2^)	63.4±76.9	37.2±35.3	74.9±67.2	68.7±87.8	P_2_ = .625 P_3_ = .744 P_4_ = .997
		p = .347	p = .726	p = 887	P_A_ = .641
Abdominal Aorta level2 Lumen Area (x10^3^ µm^2^)	669.1±195.9	545.5±154.2	620.2±141.7	558.6±157.2	P_2_ = .738 P_3_ = .998 P_4_ = .834
		p = .134	p = .530	p = .181	P_A_ = .313
Intra-plaque Elastin (%)	15.57±4.22	20.84±2.79	20.63±3.4	25.24±2.52	P_2_ = .999 P_3_ = .025 P_4_ = .018
		p = .004	p = .009	p<.001	P_A_<.001
Tunica Media Elastin (%)	34.16±4.6	36.04±6.28	36.81±4.28	33.43±3.82	P_2_ = .984 P_3_ = .626 P_4_ = .412
		p = .455	p = .199	p = .704	P_A_ = .375
Intra-plaque Collagen (%)	19.02±4.05	24.68±7.02	27.49±9	31.14±4.44	P_2_ = .765 P_3_ = .132 P_4_ = .589
		p = .040	p = .018	p>.001	P_A_ = .002
Average Total MMPstain (%)	13.90±1.65	10.23±1.44	10.01±1.31	8.89±1.40	P_2_ = .987 P_3_ = .187 P_4_ = .325
		p<.001	p<.001	p<.001	P_A_<.001
Average Total TIMP stain (%)	7.66±1.20	7.39±1.81	8.30±1.25	10.43±1.88	P_2_ = .569 P_3_ = .001 P_4_ = .022
		p = .699	p = .258	p = .001	P_A_<.001
Total MMP/TotalTIMP stain ratio	1.86±0.39	1.47±0.49	1.21±0.13	0.86±0.10	P_2_ = .278 P_3_ = .001 P_4_ = .050
		p = .072	p<.001	p<.001	P_A_<.001

Measurements are based on tissue section histochemistry. Tissues were extracted at experimental week 24, after the therapeutic interventions.

p: p value of differences in variables between each experimental group and control group; P_A_: significance of variable differences between all groups (one-way ANOVA); P_2_: significance of differences in variables between groups AT and EX (post-hoc Tuckey HSD test); P_3_: significance of differences in variables between groups AT and AT+EX (post-hoc Tuckey HSD test); P_4_: significance of differences in variables between groups EX and AT+EX (post-hoc Tuckey HSD test).

**Table 3:** Circulating concentrations of MMPs and TIMPs at the beginning and the end of the study (in ng/ml).

	Experimental week	CO (n = 10)	AT (n = 10)	EX (n = 10)	AT+EX (n = 10)	P2	P3	P4	ANOVA, post-hoc p values
MMP-2	week 1	767.7±103.8	745.3±71	765.3±80	804.9±71	.58	.954	.362	**P_5_ = .840**
	week 24	1102.7±131.6	885.2±111.4	843.4±97.3	840±107.3	.001	<.001	<.001	**P_6_ = .814**
	P1	<.001	.002	.086	.055				**P_7_>.999**
MMP-3	week 1	39.88±6.6	41.95±4.7	38.83±7	40.1±5.1	.43	.735	.950	P_5_ = .998
	week 24	52.43±8.4	48.97±9.7	49.7±7.8	46.6±9.3	.40	.465	.166	P_6_ = .937
	P1	.007	.052	.020	.095				P_7_ = .870
MMP-8	week 1	18.3±4.2	19.6±3.9	20.9±3.6	19.4±3.9	.487	.153	.564	P_5_ = .433
	week 24	23.7±3.2	20.2±5	23.430±5.4	19.2±4.7	.082	.884	.024	P_6_ = .962
	P1	.013	.775	.173	.921				P_7_ = .203
MMP-9	week 1	66.2±5	65.8±7.6	71.8±8.5	70.4±7.2	.875	.092	.159	**P_5_ = .529**
	week 24	83.9±10.5	70.6±10.5	77±14	70.8±4.8	.011	.231	.002	**P_6_>.999**
	P1	.001	.14	.367	.889				**P_7_ = .558**
TIMP-1	week 1	207.8±46.1	174.7±42.9	192.2±43.8	210.7±63.4	.114	.448	.906	P_5_ = .843
	week 24	254.2±63.9	262.7±70.9	286.5±52.3	273.5±66.9	.775	.233	.517	P_6_ = .982
	P1	.026	.01	.001	.022				P_7_ = .969
TIMP-2	week 1	263.6±19.5	276.1±37.7	279.9±49.2	279±34.9	.368	.350	.24	**P_5_ = .556**
	week 24	327.6±42.9	356.2±27.5	375.8±36.5	368.8±22.1	.093	.015	.015	**P_6_ = .832**
	P1	.004	<.001	.001	<.001				**P_7_ = .964**
TIMP-3	week 1	3.6±2.5	4.48±1.9	3.26±2.6	3.48±1.9	.883	.728	.908	P_5_ = .997
	week 24	5.19±2.2	4.97±1.9	5.14±2	6.26±1.7	.242	.960	.241	P_6_ = .467
	P1	.16	.515	.074	.003				P_7_ = .588

P1: p value of variable changes within groups at week 1 compared to week 24; P2, P3, P4: p values of differences between groups ΑΤ, EX and AT+EX respectively, against group CO on the corresponding week; P_5_: Significance of differences in variables on week 24 between groups AT and EX (post-hoc Tuckey HSD test); P_6_: Significance of differences in variables on week 24 between groups AT and AT+EX (post-hoc Tuckey HSD test); P_7_: Significance of differences in variables on week 24 between groups EX and AT+EX (post-hoc Tuckey HSD test); p value in bold: ANOVA significance value for variances on week 24 between all groups is less than 0.05.

Experimental week count started when mice were 8 weeks old, and entered high fat diet.

**Table 4:** Densitometry measurements in zymography and reverse zymography gels.

	CΟ (n = 10)	ΑΤ (n = 10)	EX (n = 10)	ΑΤ+EX (n = 10)	ANOVA, post-hoc p values
MMP-2 (pg)	81.3±11.1	61.0±8.7	100.3±12.2	81.8±10.4	P_2_<.001 P_3_ = .001 P_4_ = .004
		p<.001	p = .003	p = .931	P_A_<.001
pro-MMP-2 (pg)	102.2±12.0	83.1±12.0	86.4±10.9	81.3±13.8	P_2_ = .943 P_3_ = .988 P_4_ = .813
		p = .003	p = .009	p = .003	P_A_ = .003
MMP-9 (pg)	113.5±13.9	92.3±10.3	97.1±7.5	89.1±10.0	P_2_ = .772 P_3_ = .921 P_4_ = .395
		p = .002	p = .006	p<.001	P_A_<.001
TIMP-1 (peak area)	3002±357	3253±402	3903±562	4045±472	P_2_ = .015 P_3_ = .002 P_4_ = .897
		p = .158	p<.001	p<.001	P_A_<.001
TIMP-2 (peak area)	2356±324	1932±336	2159±307	1905±233	P_2_ = .352 P_3_ = .997 P_4_ = .258
		p = .010	p = .180	p = .002	P_A_<.006

Values for MMP-2, MMP-9 and proMMP-2 are given in units of picograms of enzyme per homogenized sample. Values for TIMP-1 and TIMP-2 are given as densitometry measurements of peak area per homogenized sample.

p: p values of differences in variables between each experimental group and control group; P_A_: significance of variable differences between all groups (one-way ANOVA); P_2_: significance of differences in variables between groups AT and EX (post-hoc Tuckey HSD test); P_3_: significance of differences in variables between groups AT and AT+EX (post-hoc Tuckey HSD test); P_4_: significance of differences in variables between groups EX and AT+EX (post-hoc Tuckey HSD test).

### Mean plaque area, collagen and elastin localization

**Figure 4:**
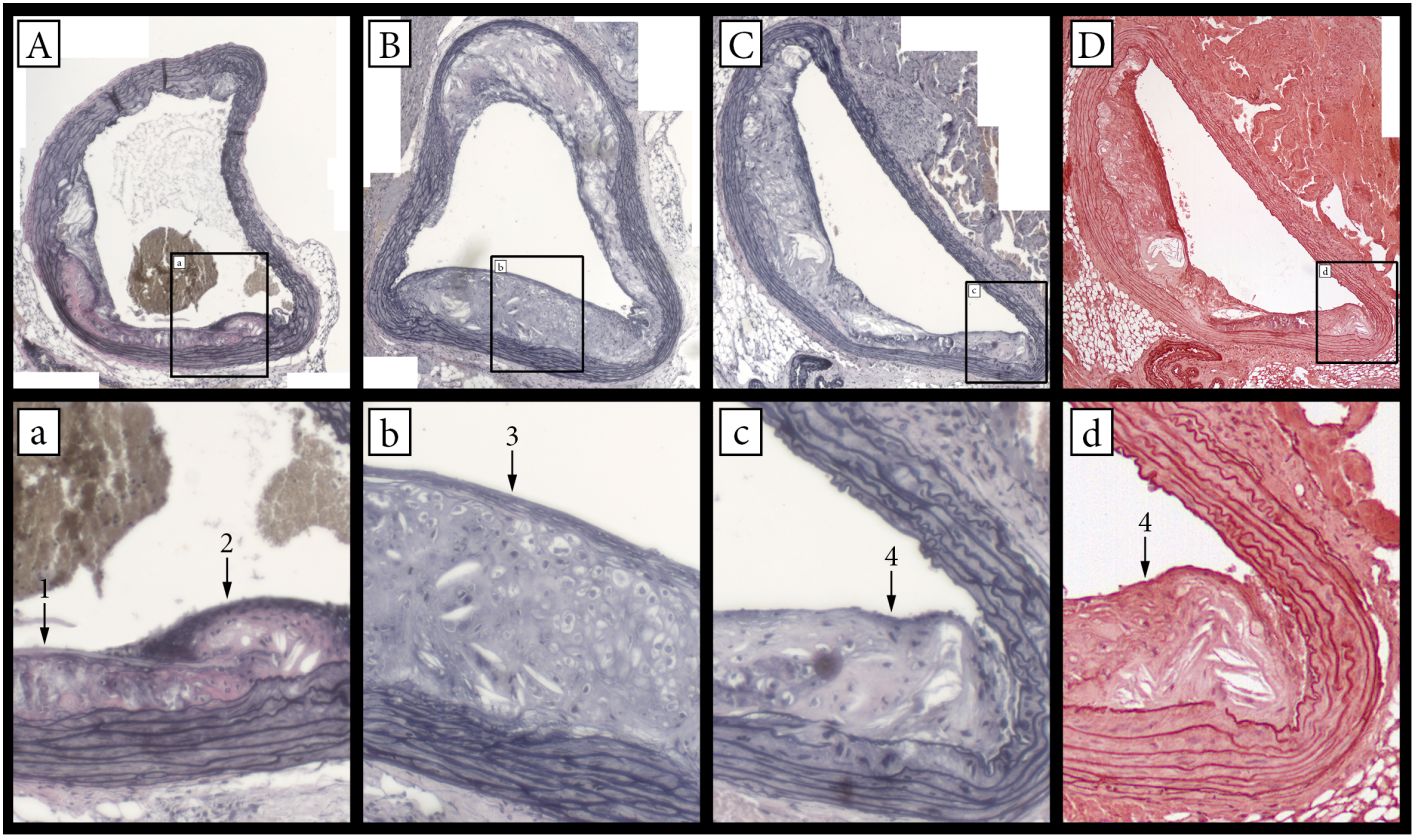
Verhoeff’s Van Gieson and Orcein stainings for the visualization of elastin content and stainings co-localization. Lowercase letter image naming corresponds to a higher magnification of the defined section in the respectively named Uppercase named image. A–C: Verhoeff’s Van Gieson staining. D: Orcein staining. 1: Little or undetectable elastin. 2: Thick amorphous elastin content. 3: Formed elastin fibers. 4: Thin amorphous elastin.

The morphometric data from sections are presented in Table 2. At the end of the study a significant decline in the mean atherosclerotic plaque area in the aortic arch was documented in all atorvastatin treated groups (group AT, group AT+EX) compared to CO group (p<.05). Similarly, the upper section of the abdominal aorta benefitted greatly by all interventions, as far as lesion size is concerned. The descending thoracic aorta and the abdominal aorta below the renal arteries displayed limited amounts on atherosclerotic lesions in the control group, as well as in the experimental groups, and thus there was no difference detected. In particular, 55% of the sections of the descending thoracic aorta were lesion free and in the sections of the abdominal aorta halfway between the renal arteries and the iliac arteries 70% of the sections were lesion free, in contrast to the sections of the aortic arch and the upper abdominal aorta were all sections displayed varying amounts of lesions. All intervention groups appeared with significantly higher content of collagen and elastin within the atherosclerotic plaques rather than the control group (p<.05). Moreover, the effect was significantly amplified in the combined intervention group (AT+EX), in comparison to the other intervention groups for the elastin content (ANOVA: p = .025 vs. AT, p = .018 vs. EX). To confirm the results obtained for elastin content by orcein staining, we also performed EVG staining to closely localized sections (Figure 4). EVG staining gave similar results (CO: 10.7%±2.8%; AT: 14.0%±2.7%, p vs CO = .017; EX: 14,74%±1.7%, p vs CO = .001; AT+EX: 17.3%±1.9%, p vs CO<.001). Performing a Pearson’s correlation test on paired variables between Orcein positive stain and EVG positive stain, yielded a correlation coefficient of r = .944 with a significance value of p<.001. Thus, we have concluded that the two stains are in strong agreement as far as intra-plaque elastin content is concerned. No significant differences in the elastin content of the lesion-free portions of the tunica media for any intervention group were detected (p>.19).

### The MMP/TIMP equilibrium

**Figure 5:**
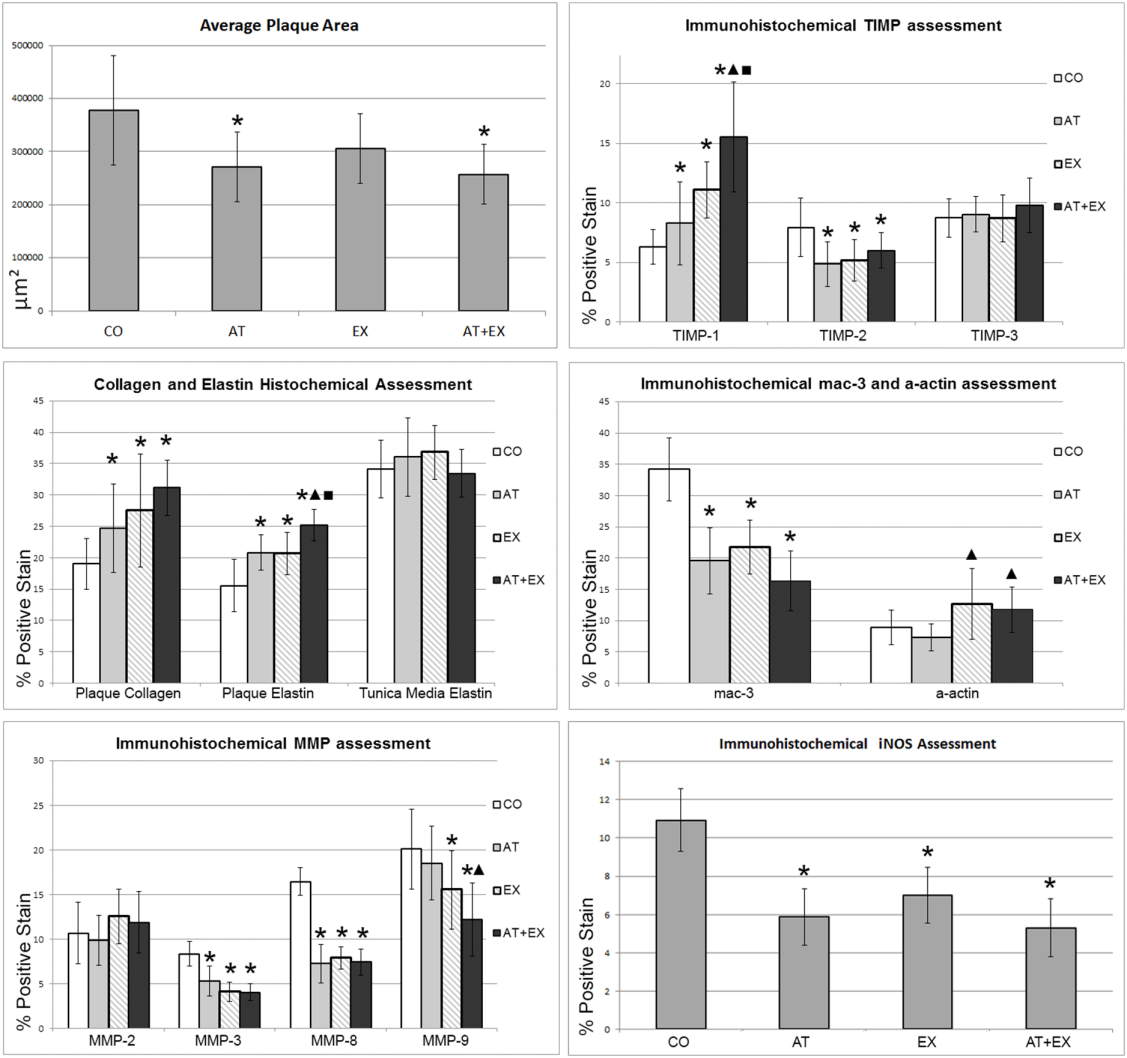
Morphometry and immunohistochemistry results for total plaque area in all examined aortic regions, collagen and elastin content, MMP, TIMP, mac-3, a-actin and iNOS intra-plaque localization and activity. *: p value vs CO group <.05; ▴: ANOVA significance value <.05 and post hoc Tuckey HSD test p value vs AT group <.05; ▪: ANOVA significance value <.05 and post hoc Tuckey HSD test p value vs EX group <.05.

The results for MMP and TIMP-1 relative concentrations within plaques are illustrated in Figure 5 and Table 2. In comparison to CO group, all intervention groups showed a significant reduction in MMP-3 (p<.001) and MMP-8 (p<.001) positive-stained area, with AT+EX group not appearing significantly differentiated compared to the other intervention groups regarding these parameters. MMP-9 relative concentrations demonstrated a significant reduction in comparison to CO group, only in the exercise treated groups (EX, AT+EX). TIMP-1 positive stain levels on the other hand, were significantly increased in all intervention groups. Moreover, combined treatment group had a significantly higher amount of TIMP-1 positive stain compared to groups AT and EX (ANNOVA: p<.001 AT vs AT+EX, p = .018 EX vs AT+EX). Compared to CO group, groups AT, EX and AT+EX demonstrated decreased levels for TIMP-2 staining, without those differences being more pronounced in the combined treatment group. MMP-2 and TIMP-3 intra-plaque levels did not show any correlation to the treatment modalities. Moreover, post-hoc analysis revealed a significant shift of total MMP to total TIMP ratio in the combined treatment group compared to the single intervention groups.

Circulating levels of the examined MMPs and TIMPs were assessed both at study entry and at the study’s end, and are presented in Table 3. MMP-2, MMP-3, MMP-8, MMP-9, TIMP-1 and TIMP-2 levels were significantly increased at the end of the study compared to baseline levels, as paired samples analysis revealed within the CO group. At the study’s end, all intervention groups exhibited significantly lower concentrations of plasma MMP-2 (AT vs CO p = 0.001, EX vs CO and AT+EX vs CO p<.001). MMP-8 was significantly reduced only in the AT+EX group (p = .024) and MMP-9 only in the atorvastatin treated groups (AT vs CO p = .011, AT+EX vs CO p = .002). While TIMP-1 revealed no differences between intervention groups and CO, TIMP-2 was significantly increased in the active groups (EX, AT+EX).

**Figure 6:**
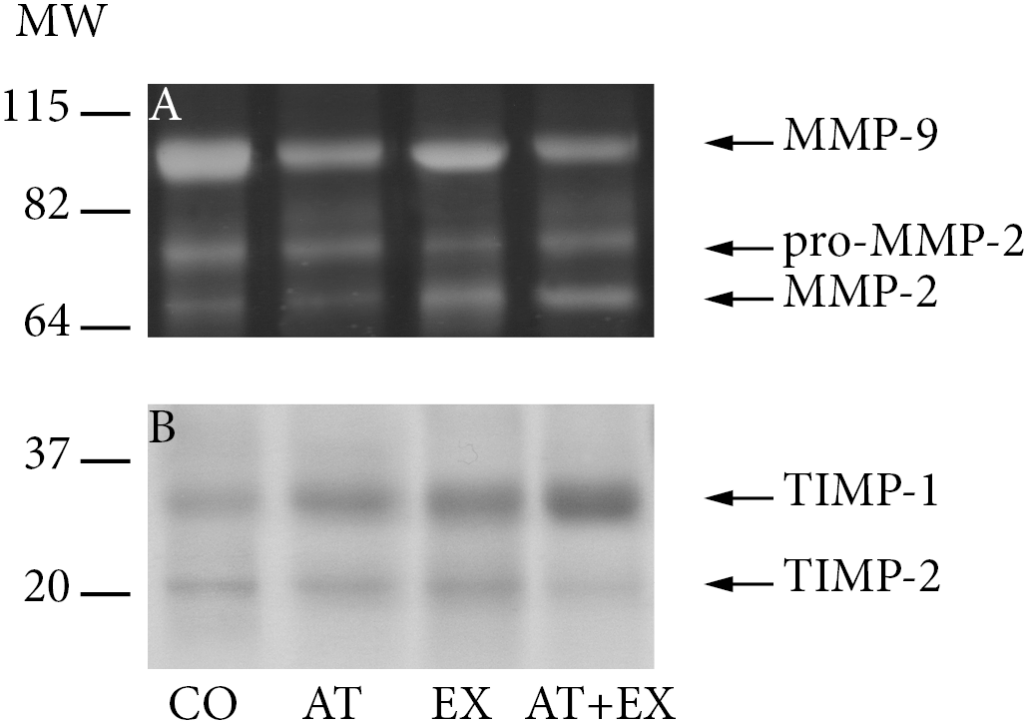
Representative images from zymograms for the identification of metalloproteinase activity and reverse zymograms for the identification of TIMP activity. A) Zymograhy, MMP-2, MMP-9 and proMMP-2 identification. B) Reverse zymography, TIMP-1 and TIMP-2 identification.

The assessment of MMP and TIMP activity within the aorta, shown in Table 4 and Figure 6 revealed a significant reduction of MMP-9 in all intervention groups compared to CO. This was accompanied by an increase of TIMP-1 activity in groups EX and AT+EX. TIMP-2 activity on the other hand, was substantially decreased in the atorvastatin treated groups. The pro-MMP-2 molecule was also detected as a consequence of the denaturation/renaturation SDS-PAGE process. While it was identified at decreased activity in all intervention groups, MMP-2 itself had a significantly lower activity in the AT group, and a higher one in the EX group.

### Macrophages and SMCs

All intervention groups showed a statistically significant reduction (p<.001) in the percentage of positive-stained area for macrophages, identified by mac-3 antigen compared to CO group (Figure 5). However, we did not detect any further differences between intervention groups. MMP-3 and MMP-8 levels closely followed the macrophage observed reduction in concentration (Pearson’s correlation coefficient r = 0.713, r = 0.775 respectfully, p<0.001 in both cases). With a less strict correlation coefficient, MMP-9 was also found to be correlated to the macrophage content (r = .328, p = .023). iNOS staining, for the identification of M1 type macrophages was correlated with the mac3 staining pattern across sections (paired samples t test, p<.05 in all groups). With respect to SMCs, all interventions failed to significantly alter the SMCs concentration in relation to control group (p>.05), as was shown by the a-actin staining within the atherosclerotic lesions.

The full data of the above described results are available in the supplementary data spreadsheet file (Data S1), as well as in the SPSS data file (Data S2).

## Discussion

In our study the 8-week interventions of exercise training, atorvastatin administration or combined treatment, remarkably attenuated the atherosclerotic stenosis progression compared to untreated mice. Furthermore, atorvastatin treatment delayed the lesion progression and all interventions promoted a more stable phenotype of the atherosclerotic lesions, as assessed by collagen, elastin, macrophages and MMP/TIMPs content. Importantly, combined treatment with atorvastatin plus exercise training conferred the most pronounced improvement in plaque stability, as measured by the lesion elastin content, despite the moderate lipid-lowering and the absence of significant changes in body-weight. In clinical trials, the two therapeutic modalities have so far produced controversial results concerning the benefit of their combined effects [18], [19]. Thus, our findings are of clinical relevance since they outline the favorable effects of pharmaceutical and non-pharmaceutical modalities on a major risk factor, that of the stability of the atherosclerotic plaque.

One of the most striking findings of the present study was the remarkable atherosclerotic lesion size amelioration in the aortic arch and the upper abdominal aorta after atorvastatin administration and the combined treatment. A number of clinical and experimental studies has produced controversial results regarding the effect of statin treatment on the lesion size [20]–[22]. The above discrepancy may be attributed to the differential effects of statin members. On the other hand, there is a lack of clinical studies demonstrating atherosclerosis regression after chronic exercise training [23]. However, exercise interventions using animal models of atherosclerosis have mostly demonstrated considerable atherosclerosis regression [24]. In our study, although exercise treatment did not achieve statistical significance neither in reducing the lesion size, nor increasing the lumen area in the aortic arch, both modalities seemed to be positively affected, as their combined per animal results yielded a significant reduction in lumen stenosis. Furthermore, lesion size was found to be positively affected in the abdominal aorta above the renal arteries (p<.05) in all intervention groups.

The anti-atherosclerotic mechanisms of statins and exercise have been partly ascribed to their well-established lipid-lowering properties [17], [25]. It is worth mentioning that our atorvastatin group modestly improved lipid profile, while the exercise-treated group did not get any benefit in lipid levels. Also, although body weight was reduced in all groups, stress related weight loss, as observed in the control group due to the placebo treatment, seems to be the predominant cause of weight loss in all intervention groups as well, because they did not sustain significantly more weight loss compared to control. So there is great possibility that other non-lipid related actions of statin and exercise treatment might have suppressed the advancement of atherosclerosis. Experimental trials have documented the inhibition of plaque development consistently after statins administration and those effects were independent of lipid alterations [26]. Similarly, other researchers have reported plaque regression in exercised apoE^−/−^ mice and those anti-atherogenic effects were mainly ascribed to other than lipid-lowering mechanisms [27]. The anti-inflammatory effects of exercise are well documented in the literature. During exercise, IL-6 is produced by muscle cells via a TNF-independent pathway. IL-6 stimulates the appearance of other anti-inflammatory cytokines such as IL-1ra and IL-10 in the circulation and inhibits the production of the proinflammatory cytokine TNF-a [28]. In our study plaque size amelioration was associated with beneficial effects on MMP/TIMP equilibrium. We postulated that the suppression of proteolytic enzymes and inflammatory cells infiltration could attenuate the plaque development.

To our knowledge, this is the first study examining the effects of combined statin and exercise treatment on atherosclerotic plaque stability. We observed the effects of atorvastatin and exercise training in most parameters of plaque vulnerability [29]. In particular, even though the two therapeutic strategies showed synergy in affecting only a limited number of examined parameters, almost all the other examined parameters of plaque vulnerability were positively affected by either one of the therapeutic interventions. Importantly, elastin positive stain showed a significant rise after combined treatment compared to each single intervention. Given the fact that elastin was primarily present in the fibrous cap of the atheromata, this additive effect contributes to an enhanced atheroma stability and resistance to rupture. In some cases, like in MMP-9 tissue distribution and TIMP-1 activity, where one therapeutic modality conferred beneficial effects while the other strategy did not seem to have a positive contribution, the combined treatment group retained the beneficial effects. As the effective strategy was not the same in all such factors, combined treatment yielded an overall more stable phenotype, not in an additive but rather in a complementary way regarding those parameters.

Taking into account the absence of significant changes in the plaque infiltration of SMCs, the cellular sources of the extracellular matrix, we hypothesized that the rise in collagen and elastin concentrations might be associated with the inhibition of their proteolytic enzymes, such as MMPs rather than their increased production. In particular, collagen and elastin increment was accompanied with significant downregulation of MMPs and considerable increase in TIMP-1 in all our active groups. The additive effect of the two therapeutic modalities concerning elastin intra-plaque content, was in accordance with a similarly addititve effect of TIMP-1 positive stain and total MMP/TIMP ratio downregulation, pointing towards an increased proteolysis inhibition as the primary cause of the observed elastin changes. Notably, MMP-9 concentrations and activity levels followed the inverse pattern of elastin concentrations. Moreover, MMP-2 activity assessment revealed differentiations in the proenzyme activation by TIMP-2. TIMP-2 can act both as an MMP inhibitor and activator, in a controversial role that is mainly concentration dependent [30]. The lower TIMP-2 concentrations were consistent with its reduced activity levels in all treatment groups. However, this possible regulatory mechanism requires further investigation. Collagen intra-plaque levels closely followed the inverse pattern of MMP-8 concentrations, strengthening the reduced proteolysis hypothesis. Previous studies have proposed the changes in SMCs phenotype after exercise training or statins as the cause of plaque stabilization [31], [32].

Another important finding of our study was the significant reduction in macrophages infiltration within the lesions of both atorvastatin and exercise-treated groups when compared to the control group. Macrophages are the predominant cellular sources of MMPs. Thus, the remarkable reduction in their concentration provides a plausible explanation for the proportional reduction in MMPs across our groups. Although proenzyme expression control is a small part of the MMP proteolytic regulation, the activation mechanisms which constitute the main regulatory factor are also macrophage dependent. Therefore, exercise seems to add anti-inflammatory benefits when it is incorporated in statin therapeutic strategy that also affect the proteolytic activity within the lesion.

It is well established that statins suppress MMPs within the atherosclerotic lesions [33]–[35]. On the other hand, limited previous studies documented the exercise-induced lowering of serum MMPs levels in high-risk individuals [36]–[38], or the reduction of MMPs concentrations within the atherosclerotic plaques in apoE^−/−^ mice [17]. In our study, both treatment modalities ameliorated MMP-8, MMP-9 and MMP/TIMP plaque relative concentrations. Moreover, the total MMP/TIMP ratio exhibited a further favorable shift in the combined treatment group compared to each of the monotherapies. MMP-8 and MMP-9 constitute emerging and well-documented, mediators of atherosclerotic plaque stability [39], [40]. The MMPs inhibitor, TIMP-1, has been documented to contribute to atherosclerotic plaque stability [41]. Therefore, the swift of MMP-9/TIMP-1 balance to less proteolytic activity strongly supports a crucial atheroprotective mechanism. Less macrophage content and suppressed MMPs activity limits the possibility of fibrous cap rupture, restricts lipid-core and further reduces inflammatory cells infiltration [42].

Increased serum levels of MMPs and TIMPs have been documented to be associated with atherosclerosis [40], [43]. Indeed, our findings support that atherosclerosis development is accompanied by an increase in MMP-2, MMP-3, MMP-8, MMP-9, TIMP-1 and TIMP-2 levels. However, an interesting finding of the present study is the fact that circulating levels of the abovementioned molecules are not correlated with their concentration within the atherosclerotic lesions after the therapeutic interventions. Therefore, while they may serve as markers of inflammation and atherosclerosis, they probably have multiple tissue origins and cannot serve as markers for the disease development or amelioration.

The main limitation of the present study was the fact that immunohistochemistry-based measurements do not directly express absolute protein activity. In addition, fibrous cap rupture or erosion is rarely observed in the atherosclerotic plaques of apoE^−/−^ mice fed western-type diet. Thus, we estimated plaque vulnerability indirectly by using a number of parameters associated with plaque texture. Furthermore, while the improved lumen stenosis factor in experimental groups could be postulated to lead to an enhanced circulatory function, such a possibility was not explored in the present study, because no functional data, like tension or flow data, were recorded during animal euthanasia. Finally, TIMPs are known to form complexes with the MMPs. These complexes inhibit the proteolytic activity of MMPs, but can also promote the activation of MMP pro-enzymes. The MMP/TIMP complexes cannot be diversified from non-complex molecules when stained, and are separated when their activity is assessed via zymography, so we could not examine their precise role in plaque stability. Instead, we searched for co-localization, which enabled us for mapping their activity in the plaque.

In conclusion, both atorvastatin and exercise enhanced plaque stability in apoE-deficient mice, without significant change in body-weight. Our results were associated with favorable effects on inflammatory (macrophages) and pro-atherogenic (MMP-3, MMP-8, MMP-9) or anti-atherogenic (TIMP-1) mediators. Moreover, simultaneous treatment with atorvastatin and exercise yielded more beneficial results than each intervention alone. Our findings outline the importance of pharmaceutical intervention accompanied with lifestyle alteration in the management of the atherosclerotic complications.

## Supporting Information

Figure S1 **Sirius red stains of serial sections of the aortic arch in Apo-E−/− mouse for the visualization of collagen content.** Section thickness was set at 5 µm and original magnification at 100x. A: Group CO. B: Group AT. C: Group EX. D: Group AT+EX.
(TIF)

Figure S2 **Orcein stains of serial sections of the aortic arch in Apo-E−/− mouse for the visualization of elastin content.** Section thickness was set at 5 µm and original magnification at 100x. A: Group CO. B: Group AT. C: Group EX. D: Group AT+EX.
(TIF)

Figure S3 **Anti-MMP-8 immunohistochemisrty stains of serial sections of the aortic arch in Apo-E−/− mouse for the visualization of MMP-8 content.** Section thickness was set at 5 µm and original magnification at 100x. A: Group CO. B: Group AT. C: Group EX. D: Group AT+EX.
(TIF)

Figure S4 **Immunohistochemistry stains and negative control in co-localized sections.** A: anti-MMP8 stain. B: anti-TIMP-1 stain. C: negative control. Original magnification 400x.
(TIF)

Data S1 **Spreadsheet file (Microsoft Excel v.14.0) containing the full data set of the study’s described and discussed results.**
(XLSX)

Data S2 **Statistical analysis file (IBM SPSS v.20) containing the full data set of the study’s described and discussed results.**
(SAV)

Protocols S1 **Detailed description of the experimental protocols that were performed in this study.**
(DOCX)
