# Supplementary figures and images for: The Complementary Effects of Atorvastatin and Exercise Treatment on the Composition and Stability of the Atherosclerotic Plaques in ApoE Knockout Mice
Source: PLoS One. 2014 Sep 29;9(9):e108240. doi: 10.1371/journal.pone.0108240 (PMC4180453; doi:10.1371/journal.pone.0108240)

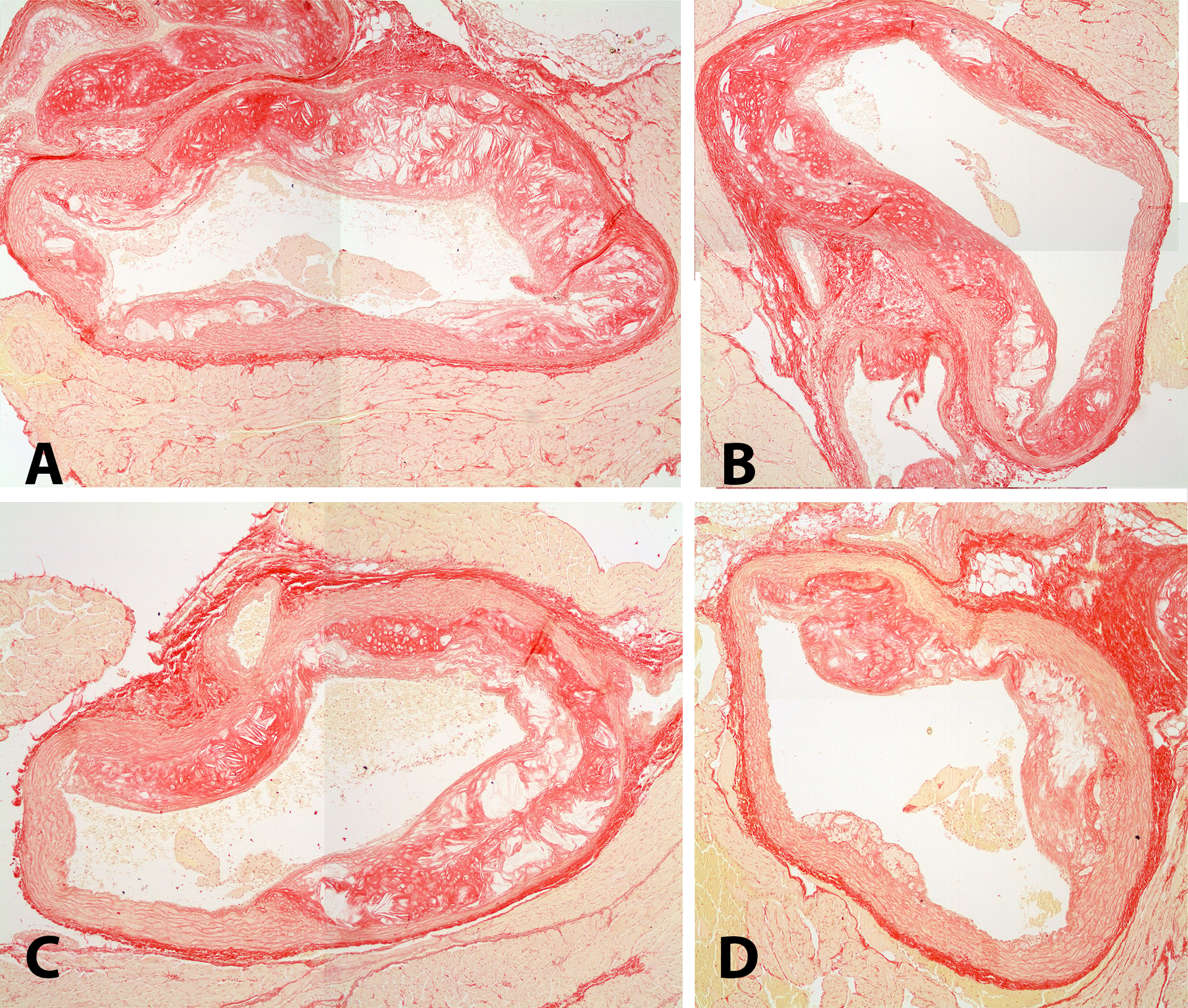

Supplement: Figure S1 — Sirius red stains of serial sections of the aortic arch in Apo-E−/− mouse for the visualization of collagen content. Section thickness was set at 5 µm and original magnification at 100x. A: Group CO. B: Group AT. C: Group EX. D: Group AT+EX. (TIF) [file pone.0108240.s001.tif]

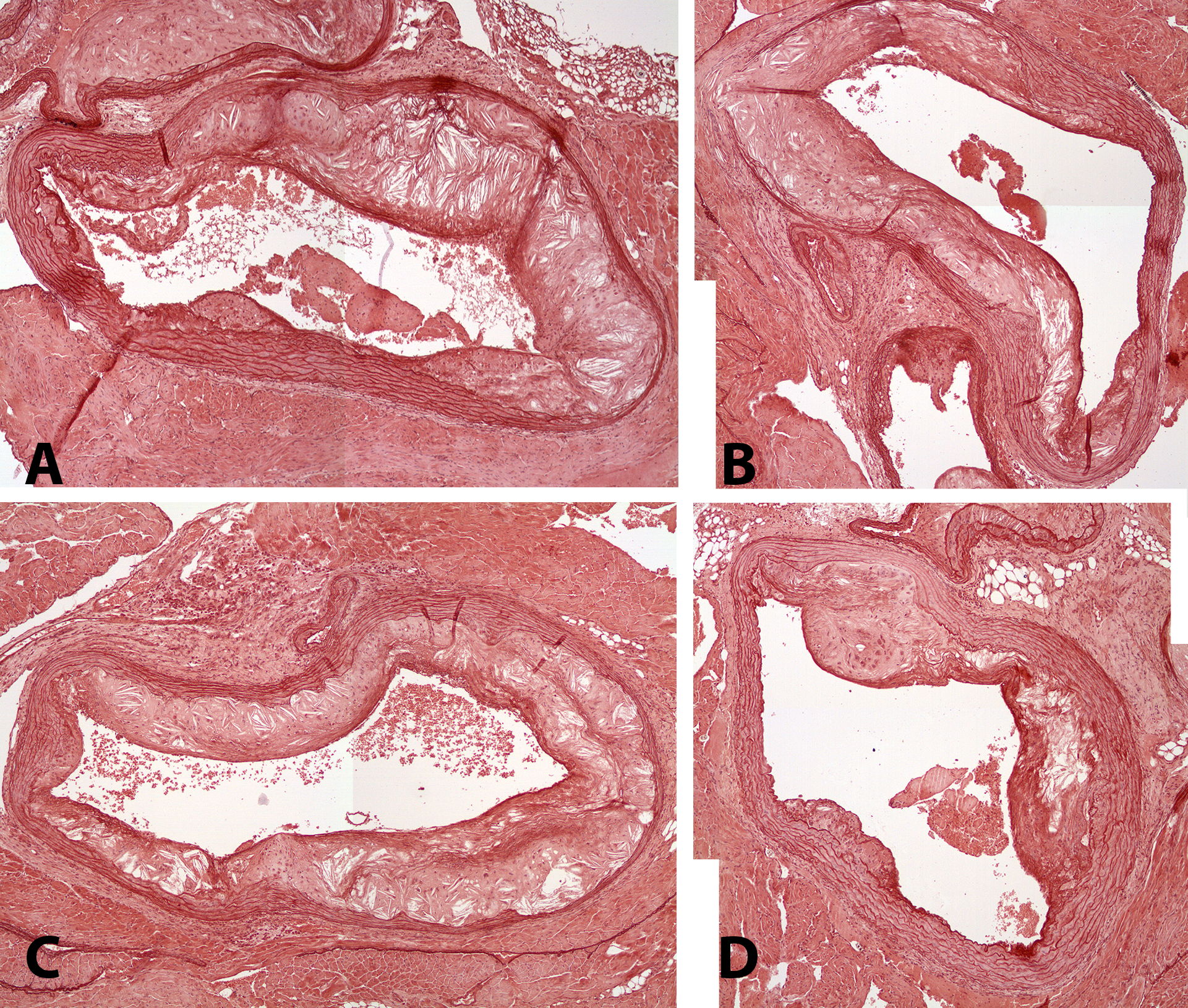

Supplement: Figure S2 — Orcein stains of serial sections of the aortic arch in Apo-E−/− mouse for the visualization of elastin content. Section thickness was set at 5 µm and original magnification at 100x. A: Group CO. B: Group AT. C: Group EX. D: Group AT+EX. (TIF) [file pone.0108240.s002.tif]

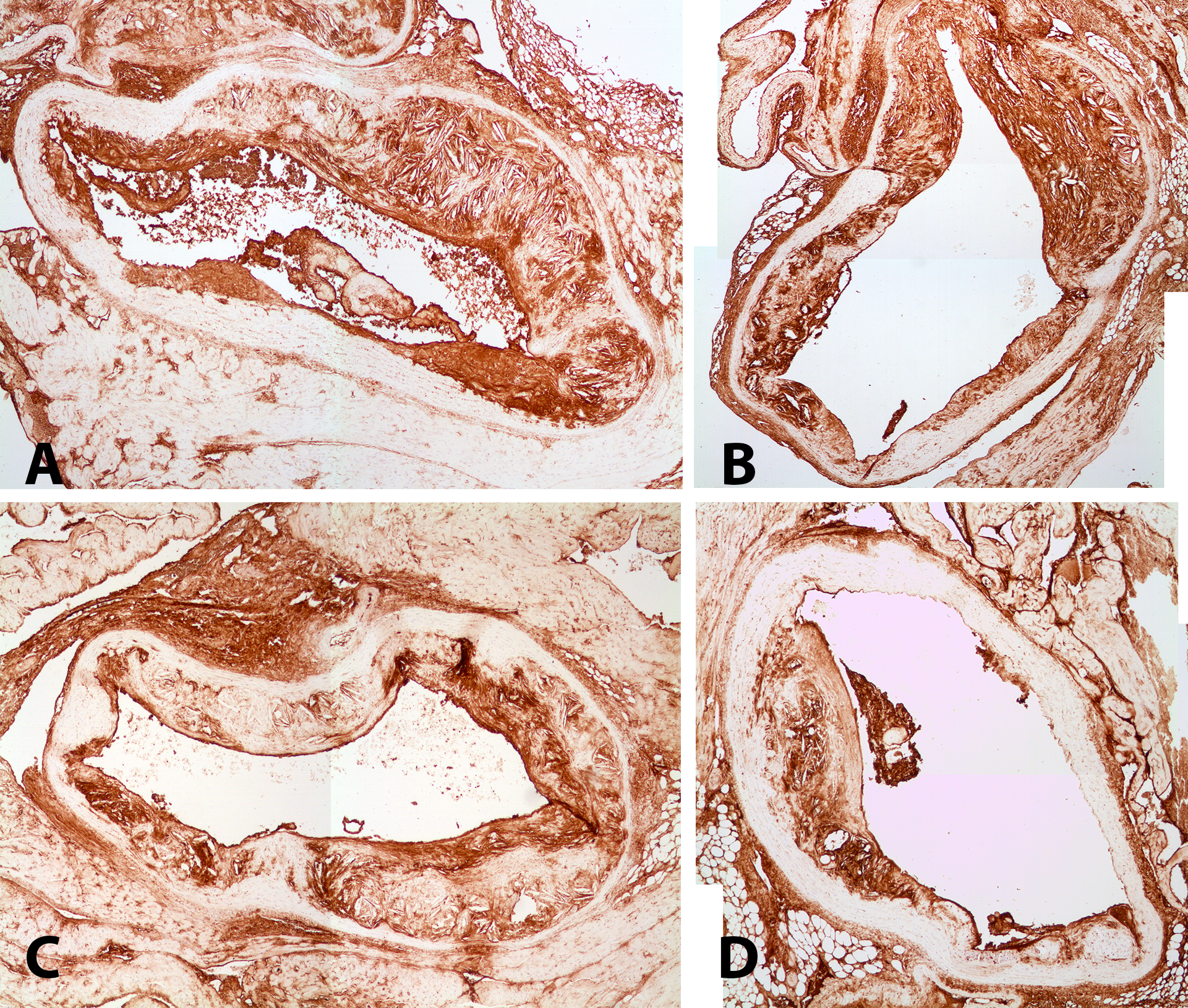

Supplement: Figure S3 — Anti-MMP-8 immunohistochemisrty stains of serial sections of the aortic arch in Apo-E−/− mouse for the visualization of MMP-8 content. Section thickness was set at 5 µm and original magnification at 100x. A: Group CO. B: Group AT. C: Group EX. D: Group AT+EX. (TIF) [file pone.0108240.s003.tif]

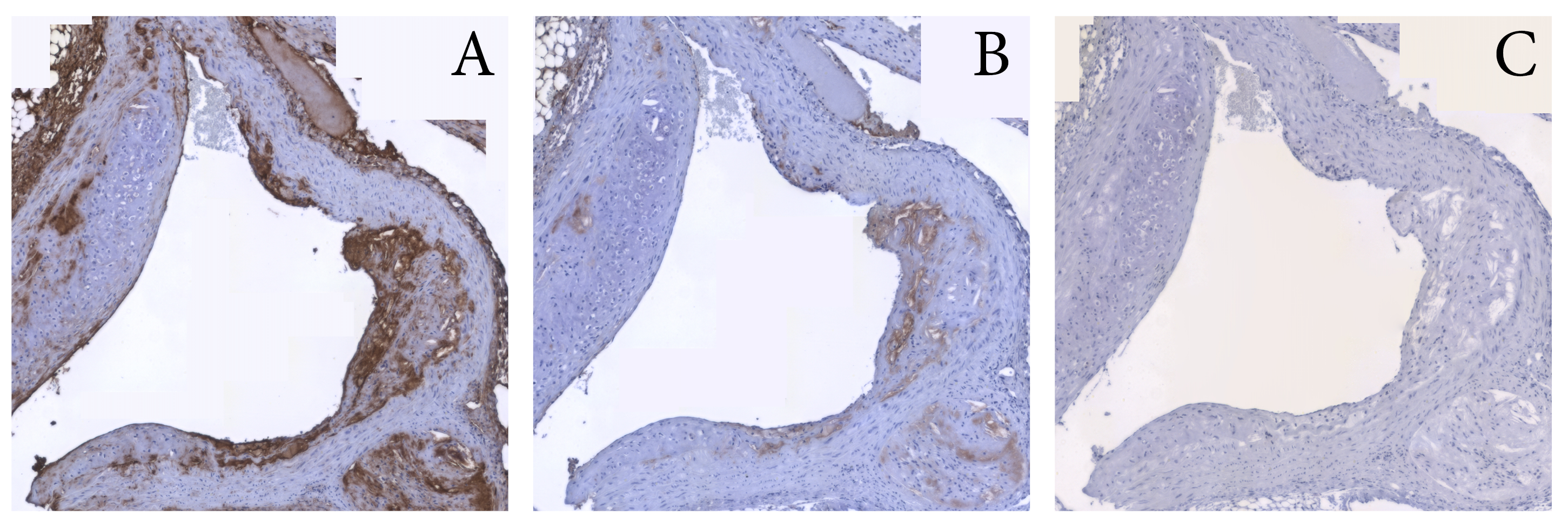

Supplement: Figure S4 — Immunohistochemistry stains and negative control in co-localized sections. A: anti-MMP8 stain. B: anti-TIMP-1 stain. C: negative control. Original magnification 400x. (TIF) [file pone.0108240.s004.tif]
