# Supplementary material for: The Complementary Effects of Atorvastatin and Exercise Treatment on the Composition and Stability of the Atherosclerotic Plaques in ApoE Knockout Mice
Source: PLoS One. 2014 Sep 29;9(9):e108240. doi: 10.1371/journal.pone.0108240 (PMC4180453; doi:10.1371/journal.pone.0108240)
Supplement: Protocols S1 — Detailed description of the experimental protocols that were performed in this study. (DOCX) [file pone.0108240.s007.docx]

**Detailed protocols utilized**

**Animal euthanasia**

Inhaled isoflurane

Total blood acquisition via heart puncture.

**Tissue samples acquisition**

Heart along with aortic arch in formalin 10% solution for paraffin embedding. Part of the aortic arch was snap frozen using isopantane and stored at -80^o^C.

Total blood centrifugation at 2000rpm for 15min (Heraeus Megafuge 16R; Thermo Fisher Scientific, Waltham, MA, USA)

**Tissue fixation - embedding**

For paraffin embedding (paraffin sections):

In formalin 10% solution @ 4^o^C overnight

Wash well with tap water

Place in 70% EtOH @ 4^o^C overnight

80% EtOH for 1 hour

95% EtOH for 1 hour

100% EtOH (A) for 1 hour

100% EtOH (B) for 1 hour

Xylene (A) for 1 hour

Xylene (B) for 1 hour

Parafin (A) @ 60^o^C for 1 hour

Parafin (B) @ 60^o^C for 1 hour

Tissue placement in paraffin molds and sample plastic rings

Store @ -20^o^C

**Tissue sectioning**

Section thickness: 5μm

RM2255 - Fully Automated Rotary Microtome; Leica Biosystems GmbH, Wetzlar, Germany

Consecutive sections

3 sections per slide (poly-L lysine slides; Thermo Scientific, Gerhard Menzel GmbH, Braunschweig, DE)

Sections in each slide were 50μm apart in tissue (1 every 10 sections)

**Sections H+E staining**

Parafin melting by placement of slides on thermoblock @ 60^o^C for 5min.

Deparafinization in 2 consecutive xylene immersions 10min each.

Hydrophilization by immersion in EtOH 100% for 5min.

Gradual hydration by immersion in 95%ΕtΟΗ, 80%ΕtΟΗ, 50%EtOH, tap water, 5min each.

Immersion in Harris Hematoxylin solution (351946T - Hamatoxylin Harris (mercury free); VWR

Radnor, PA, USA) for 60sec

Wash in tap water. (x3)

Immersion in Eosin alcoholic solution (RRSP37/E - Eosin sln 1%; Biostain Ready Reagents Ltd. Manchester, UK) for 60sec.

Dips in tap water (x3)

Gradual dehydration by immersion in 80%ΕtΟΗ, 95%ΕtΟΗ, 100% EtOH (A), 100% EtOH (B), 30sec each.

Immersion in xylene 100% for 5min. (x2)

Mount with DPX (36029 DPX mountant; VWR Radnor, PA, USA) and coverslip (631-0146 - coverslips 24x50; VWR Radnor, PA, USA)

Let Dry for 24hr.

**Sections Sirius Red staining**

Parafin melting by placement of slides on thermoblock @ 60^o^C for 5min.

Deparafinization in 2 consecutive xylene immersions 10min each.

Hydrophilization by immersion in EtOH 100% for 5min.

Gradual hydration by immersion in 95%ΕtΟΗ, 80%ΕtΟΗ, 50%EtOH, tap water, 5min each.

Immersion in picro-sirius red solution* for 60min.

Dips in acidified water*, (10 sec)

Dips in tap water (x3)

Gradual dehydration by immersion in 80%ΕtΟΗ, 95%ΕtΟΗ, 100% EtOH (A), 100% EtOH (B), 30sec each.

Immersion in xylene 100% for 5min. (x2)

Mount with DPX (36029 DPX mountant; VWR Radnor, PA, USA) and coverslip (631-0146 - coverslips 24x50; VWR Radnor, PA, USA)

Let Dry for 24hr.

* picrosirius red solution: In 250ml ddH2O dilute picric acid until saturation.

add 250μg sirius red powder.

Add small amount of picric acid to verify saturation

* acidified water solution: In 248,5ml dH_2_O add 1,25ml anhudrous citric acid

**Sections Orcein staining**

Parafin melting by placement of slides on thermoblock @ 60^o^C for 5min.

Deparafinization in 2 consecutive xylene immersions 10min each.

Hydrophilization by immersion in EtOH 100% for 5min.

Gradual hydration by immersion in 95%ΕtΟΗ and 80%ΕtΟΗ 5min each.

Immersion in alcoholic orcein solution* for 15min @ 37^o^C on stirrer.

Wash by dipping in EtOH 70% solution.

Gradual dehydration by immersion in 80%ΕtΟΗ, 95%ΕtΟΗ, 100% EtOH (A), 100% EtOH (B), 30sec each.

Immersion in xylene 100% for 5min. (x2)

Mount with DPX (36029 DPX mountant; VWR Radnor, PA, USA) and coverslip (631-0146 - coverslips 24x50; VWR Radnor, PA, USA)

Let Dry for 24hr.

* alcoholic orcein solution: Dilute 2,5gr orcein in 250 ml EtOH 70%. Stir under heating @ 37^o^C for 10min. Filtering and addition of 1ml HCl 30%

**Sections Verhoeff’s van geison staining**

Parafin melting by placement of slides on thermoblock @ 60oC for 5min.

Deparafinization in 2 consecutive xylene immersions 10min each.

Hydrophilization by immersion in EtOH 100% for 5min.

Gradual hydration by immersion in 95%ΕtΟΗ, 80%ΕtΟΗ, 50%EtOH, tap water, 5min each.

Stain in modified Verhoeff at room temperature for 7 minutes or in a 60 °C oven for 3 1/2 minutes.
Wash in running warm tap water for 1 minute.

Differentiate in Ferric Chloride, 0.4% aq. for 75 seconds.

Wash in running warm tap water for 5 minutes.

Counterstain in Van Geisons solution for 60 seconds.

Dehydrate, clear and mount with Permount

Verhoeff Working Elastic solution:
•  3 parts Alcoholic Hematoxylin, 3%
•  2 parts Ferric Chloride 2%, aqueous
•  1 part Lugol's Iodine

mix just before use, in the particular order, and with good mixing between additions.

Van Geison’s solution:

1.0 ml 1% Acid fuchsin

45.0 ml saturated Picric acid aqueus solution

Mix well. Allow to stand overnight

**Section IHC staining**

Parafin melting by placement of slides on thermoblock @ 60^o^C for 5min.

Deparafinization in 2 consecutive xylene immersions 10min each.

Hydrophilization by immersion in EtOH 100% for 5min.

Gradual hydration by immersion in 95%ΕtΟΗ, 80%ΕtΟΗ, 50%EtOH, tap water, 5min each.

Antigen retrieval by immersion in Citrate buffer solution 10mM pH=6,0 0,1% Tween20. Boil in microwave @ 580wt for 20min. Add Citrate buffer appropriately, as boiling buffer evaporates, for the sections not to immersed and not dry.

Let cool @ RT (approx. 30min).

Wash in PBS (Phosphate Buffer Saline) pH=6,0 for 5min. (x3)

Block endogenous peroxidase by immersing in H_2_O_2_ 1,5% methanol solution for 15min in darkness.

Wash in PBS for 5min. (x3)

Biotin and Avidin blocking using Vector Laboratories Biotin-Avidin blocking kit (SP-2001)

Blocking of nonspecific epitopes with blocking serum (FBS 10% in PBS, 0,1% Tween20) for 40min.

Drain blocking serum without washing.

Apply primary antibody in appropriate dilution (see table next) in PBS 0,1% Tween20.

Incubate in moisture chamber @ 4^ο^C o/n.

Apply secondary biotynylated antibody (VECTASTAIN Elite ABC Kit; PK-6200) for 2 hours @ RT

Wash in PBS for 5min. (x3)

Apply streptavidin complex for 20min (VECTASTAIN Elite ABC Kit; PK-6200) @ RT

Wash in PBS for 5min. (x3)

Stain with DAB solution for 2min

Stop reaction by immersing in dH_2_O

Counterstain with Harris hematoxylin for 30sec

Dips in tap water (x3)

Gradual dehydration by immersion in 80%ΕtΟΗ, 95%ΕtΟΗ, 100% EtOH (A), 100% EtOH (B), 30sec each.

Immersion in xylene 100% for 5min. (x2)

Mount with DPX (36029 DPX mountant; VWR Radnor, PA, USA) and coverslip (631-0146 - coverslips 24x50; VWR Radnor, PA, USA)

Let Dry for 24hr.

Negative control stainings were performed in one section in every slide (slides contained 3 sections each). In the negative control sections, the primary antibody solution was replaced by blocking serum.

Tween 20: P9416 - TWEEN® 20 viscous liquid; Sigma-Aldrich St. Louis, MO, USA

FBS: Vector Laboratories Inc. Burlingame, CA, USA

DAB solution: SK-4100 - DAB Peroxidase Substrate Kit, 3,3’-diaminobenzidine; Vector Laboratories Inc. Burlingame, CA, USA

Haematoxylin Harris: 351946T - Hamatoxylin Harris (mercury free); VWR, Radnor, PA, USA

| Anti MMP-2 | JM-3522-100 - anti-MMP-2 | used concentration 2ng/ml |
| --- | --- | --- |
| Anti MMP-3 | JM-3523-100 - anti-MMP-3 | used concentration 2ng/ml |
| Anti MMP-8 | JM-3528-100 - anti-MMP8 | used concentration 2ng/ml |
| Anti MMP-9 | 5980-0911 | used concentration 1,5ng/ml |
| Anti TIMP-1 | DY990921 - ΜΑΒ-ΤΙΜΡ-1 | used concentration 3ng/ml |
| Anti TIMP-2 | AF7010 - TIMP2 antibody | used concentration 3ng/ml |
| Anti a-actin | CM079A-032607 | used concentration 2ng/ml |
| Anti Mac-3 | 550292 - Mac-3 | used concentration 1,5ng/ml |
